# Identification of PSMD7 as a prognostic factor correlated with immune infiltration in head and neck squamous cell carcinoma

**DOI:** 10.1042/BSR20203829

**Published:** 2021-03-24

**Authors:** Si Zhang, Siwei Yu, Jiulong Wang, Zhigang Cheng

**Affiliations:** Department of Oral and Maxillofacial Surgery, The Central Hospital of Wuhan, Tongji Medical College, Huazhong University of Science and Technology, Wuhan, China

**Keywords:** HNSCC, immune infiltration, OS, prognostic factor, PSMD7

## Abstract

**Background:** Recurrent locally advanced or metastatic head and neck squamous cell carcinoma (HNSCC) is associated with dismal prognosis because of its highly invasive behavior and resistance to conventional intensive chemotherapy. The identification of effective markers for early diagnosis and prognosis is important for reducing mortality and ensuring that therapy for HNSCC is effective. Proteasome 26S subunit, non-ATPase 7 (PSMD7) is an ATP-independent component of the 19S regulatory subunit. The prognostic value of PSMD7 and the association with immune infiltration in HNSCC remains unclear.

**Methods:** The Sangerbox, Oncomine, UALCAN and Human Protein Atlas (HPA) databases were used to examine PSMD7 expression profiles in HNSCC. The CVCDAP was used to analysis the association of PSMD7 with the prognosis of patients with HNSCC. The mechanism was investigated with gene set enrichment analysis (GSEA). The association between expression of PSMD7 and immune infiltration in HNSCC was investigated using the Tumor Immune Estimation Resource (TIMER), TISIDB database and CIBERSORT algorithm.

**Results:** PSMD7 expression was significantly up-regulated in HNSCC compared with relative normal tissues. In addition, up-regulated PSMD7 expression was associated with various clinicopathological parameters. High expression of PSMD7 suggested inferior survival of HNSCC patients. GSEA and CERES score indicated that PSMD7 was closely correlated with tumor-related signaling pathways and cell survival. Functional analyses revealed that PSMD7 was positively correlated with various infiltration levels. Moreover, PSMD7 influenced the prognosis of HNSCC patients partially via immune infiltration.

**Conclusion:** Our findings suggest that PSMD7 is associated poor prognosis in patients with HNSCC and plays an important role in tumor-related immune infiltration.

## Introduction

Head and neck cancer encompasses a series of malignances which generate in the mucosal surfaces of the upper aerodigestive tract, including the oral cavity, pharynx, larynx, and paranasal sinuses, as well as cancers of the major and minor salivary glands. The most frequent type of head and neck cancer is squamous cell carcinoma (HNSCC) [1]. Despite advances in diagnosis, surgery, chemotherapy and radiotherapy, HNSCC remains a highly malignant cancer with poor survival outcomes due to chemoradiotherapeutic resistance and metastasis [2]. Therefore, it is urgent to identify novel therapeutic targets and biomarkers for HNSCC.

Most cellular proteins are degraded through the ubiquitin–proteasome system, which is involved in a variety of biological processes. The 26S proteasome is a multisubunit complex and is composed of two 19S regulatory particles and a 20S core particle [3]. Importantly, the 26S proteasome plays an essential role in the process of degrading the substrates marked by polyubiquitin chains [4,5]. Many studies have shown that the 26S proteasome is involved in apoptosis, cell cycle progression, transcription, antigen presentation, protein quality control, DNA repair, and protein folding [6–8]. Inhibiting the proteasome function has become an important strategy for anticancer therapy since the 26S proteasome plays the critical role in cell biological processes, especially in tumor cell growth and survival [9]. Bortezomib, which can induce cell cycle arrest and apoptosis by disrupting various signaling pathways and NF-kB function in multiple myeloma, has emerged as an effective proteasome inhibitor for clinical treatment [10,11].

Proteasome 26S subunit, non-ATPase 7 (PSMD7, Rpn8, Mov34), an ATP-independent component of the 19S regulatory subunit, forms the heterodimers with PSMD14 as a functional complex which is extremely critical for degradation of ubiquitinated substrate with the proteasome [12]. PSMD7 arrests cell cycle in the G_2_/M phase during HIV infection [13]. The polymorphism rs17336700 of the PSMD7 gene is considered to be linked with ankylosing spondylitis [14]. Knockdown of PSMD7 inhibits tumorigenesis and induces cell apoptosis in esophageal squamous cell carcinoma (ESCC) via the mTOR/p70S6K pathway [15]. PSMD7 is found highly expressed in breast cancer and positively associated with poor survival. Down-regulated PSMD7 inhibits the expression of key cell cycle-related proteins and promotes the stability of p21 and p27 in breast cancer cells [16]. However, the expression profiles and prognostic value of PSMD7 in patients with HNSCC and its association with clinical characteristics and immune infiltration remain largely unknown.

In the present study, we explored the expression level of PSMD7 in HNSCC using the Cancer Genome Atlas (TCGA) and Oncomine databases. Associations between expression of PSMD7 and clinicopathological features were investigated. Moreover, the prognostic value of PSMD7 expression in HNSCC was determined by using the TCGA-HNSC dataset. In addition, the correlation between PSMD7 in HNSCC and immune cell infiltration was analyzed with the Tumor Immune Estimation Resource (TIMER) database, TISIDB and CIBERSORT algorithm. Biological function analysis of PSMD7 was performed by Gene Set Enrichment Analysis (GSEA) software. Our results shed light on the important role of PSMD7 on HNSCC and suggest a potential association between PSMD7 and tumor immune infiltration as well as an underlying mechanism.

## Methods

### TCGA datasets analysis

TCGA datasets and associated clinicopathological information were downloaded from the public database cBioportal (http://www.cbioportal.org/). The expression of PSMD7 in pan carcinoma and associated normal tissues and survival evaluation based on PSMD7 expression was performed by Sangerbox (http://sangerbox.com/), a comprehensive tool for bioinformatics analysis based on R. The clinical information from 502 patients with HNSCC for correlation analysis was retrieved. The Cox regression analyses were performed for overall survival (OS), progression-free interval (PFI) and disease-specific survival (DSS) within the CVCDAP (https://omics.bjcancer.org/cvcdap/home.do). The Kaplan–Meier plotter (www.kmplot.com) was used to analyze the prognostic values of HNSCC patients based on various clinicopathological features. To analyze the survival events, total cases were divided into two groups automatically based on getting an available outcome with computer.

### UALCAN database analysis

UALCAN is an interactive web portal which provides handy operation for performing in-depth analyses of TCGA gene expression data (http://ualcan.path.uab.edu). PSMD7 expression was determined using the ‘head and neck squamous cell carcinoma’ dataset with the ‘Expression Analysis’ module. By using the UALCAN database, the expression profiles of PSMD7 in normal and HNSCC samples based on clinicopathological parameters, such as cancer stage, age, race and tumor grade were also analyzed. The cutoff *P*-value was set as 0.05.

### Oncomine database analysis

Oncomine database, which contains 715 gene expression dataiisets with 86733 cancer and normal tissues, is currently the world’s largest oncogene chip database and integrated data mining platform (http://www.oncomine.org). PSMD7 expression in HNSCC tissues and relative normal tissues was analyzed by using the Oncomine database. The thresholds were restricted as followed: *P*-value <0.05; fold change: 1.2; gene rank: all; and data type: mRNA. Student’s *t*-test was adopted to evaluate the difference of PSMD7 expression in HNSCC and normal tissue.

### Immunohistochemistry analysis

The immunohistochemistry (IHC) staining data of protein expression and distribution of PSMD7 in normal oral epithelial and tumor tissues were obtained from the Human Protein Atlas (HPA) database (https://www.proteinatlas.org/). The PSMD7 antibodies used for IHC were from Sigma–Aldrich (HPA056069) and Santa Cruz Biotechnology (CAB019379).

### GSEA of PSMD7

GSEA with the annotation of Hallmark gene sets was performed to evaluate the association between mRNA expression of PSMD7 and variation of associated signaling pathways HNSCC. GSEA software was obtained from the Broad Institute (http://www.broad.mit.edu/gsea).

### GeneMANIA analysis

GeneMANIA (http://www.genemania.org) provides predication of protein interaction and develops an interactive functional-association network which contains a list of genes with similar functions. In the present study, we used GeneMANIA to construct a gene–gene interaction network for PSMD7 to evaluate the functions of these genes.

### Protein–protein interaction network analysis

The protein–protein interaction (PPI) network was performed using STRING (https://string-db.org/). PSMD7 (protein name) and *Homo sapiens* (organism) were chosen.

### CERES Scores of Project Achilles for PSMD7

Project Achilles systematically identifies and catalogs gene essentiality across hundreds of genomically characterized cancer cell lines. CERES score evaluating the importance of PSMD7 for cell survival of HNSCC was downloaded from Depmap portal (https://depmap.org/portal). CERES score approach to 0 means the gene is not an essential gene for cell survival, while score approach to −1 means the gene is an essential gene.

### TIMER database analysis

TIMER (https://cistrome.shinyapps.io/timer/) is a comprehensive web tool for the systematic analysis of immune infiltrates in diverse cancer types. TIMER ‘gene’ module was used to analyze correlations between PSMD7 mRNA expression and the infiltration of immune cells, including B cells, CD4^+^ T cells, CD8^+^ T cells, neutrophils, macrophages and dendritic cells in HNSCC. Additionally, the TIMER database was used to explore the association between different immune gene marker sets of immune cells and PSMD7 expression.

### Immune cell infiltration with the CIBERSORT algorithm

CIBERSORT (https://cibersort.stanford.edu/) is an established computational resource based on convolution method. Through analyzing a validated leukocyte gene signature matrix containing 547 genes and 22 human immune cell subpopulations, CIBERSORT was used to characterize the immune cell composition. These immune cell subpopulations included memory B cells, naïve B cells, seven types of T cells, plasma cells, activated natural killer (NK) cells, monocytes, resting NK cells, three types of macrophages, activated dendritic cells, resting dendritic cells, activated mast cells, resting mast cells, eosinophils and neutrophils. The present analysis evaluated the proportions of 22 types of tumor-infiltrating immune cells with CIBERSORT and assessed correlations between PSMD7 expression and the immune cell subpopulation in HNSCC. The restriction: *P*<0.05 was set to select lymphocytes that were possibly affected by PSMD7 expression. For survival analysis, the proportion < total proportion was considered as decreased group; otherwise, the group was enriched group.

### TISIDB database analysis

TISIDB is a web portal for tumor and immune system interaction that integrates multiple heterogeneous data types [26] and can evaluate correlations between the target genes and lymphocytes. The TISIDB database was employed to analyze the correlation between PSMD7 expression and 28 tumor-infiltrating lymphocytes in HNSCC.

### Statistical analysis

The hazard ratio (HR) with *P*-value was used to evaluate the significance of survival. Pearson’s correlation, Spearman’s correction and statistical significance were used to assess the association of gene expression, and the strength of the correlation was determined using the absolute values. The results were considered as statistically significance at *P<0.05, **P<0.01 and ***P<0.001.

## Results

### Expression of PSMD7 in HNSCC

**Figure 1:**
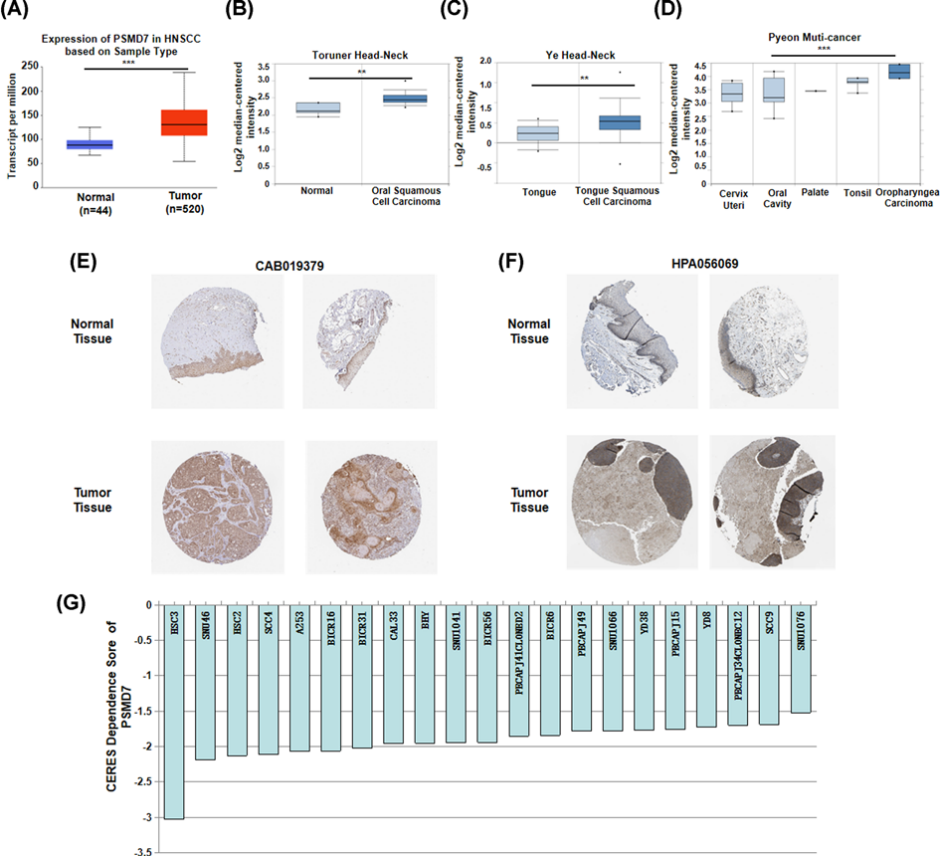
The expression of PSMD7 was higher in HNSCC (**A**) Expression of PSMD7 in HNSCC tissues compared with that in normal tissues from the UALCAN database. Box plots comparing PSMD7 expression in HNSCC patients and normal individuals were obtained from the analysis of Toruner Head-Neck (**B**), Ye Head-Neck (**C**) and Pyeon Muti-cancer (**D**) in Oncomine database. The protein expression of PSMD7 expression in HNSCC tissues and normal tissues were detected in the HPA database. IHC staining was performed by using (**E**) CAB019379 and (**F**) HPA056069 antibodies. CERES score was used to evaluate the importance of PSMD7 for cell survival of HNSCC. (**G**) CERES score approach to 0 means the gene is not an essential gene for cell survival, while score approach to −1 means the gene is an essential gene. **, *P*<0.01; ***, *P*<0.001.

PSMD7 was examined in various cancers with the Sangerbox database. As shown in Supplementary Figure S1, the mRNA expression of PSMD7 was significantly increased in several common cancer datasets, including bladder urothelial carcinoma (BLCA), Breast Invasive Carcinoma (BRCA), Cholangiocarcinoma (CHOL), Colon Adenocarcinoma (COAD), esophageal carcinoma (ESCA), Head-Neck Squamous Cell Carcinoma (HNSC), Kidney Chromophobe (KICH), Kidney Renal Clear Carcinoma (KIRC), Kidney Renal Papillary Carcinoma (KIRP), Liver Hepatocellular Carcinoma (LIHC), Lung Adenocarcinoma (LUAD), Lung Squamous Cell Carcinoma (LUSC), Rectum Carcinoma (READ), Stomach Adenocarcinoma (STAD) and Thyroid Carcinoma (THCA) datasets. The expression of PSMD7 of TCGA-HNSC in normal and tumor tissue was shown in Figure 1A. Further data mining in the Oncomine database revealed that PSMD7 was highly expressed in oral squamous cell carcinoma (Figure 1B), tongue squamous cell carcinoma (Figure 1C) and oropharyngeal carcinoma (Figure 1D) compared with relative normal tissue.

The protein expression of PSMD7 in HNSCC was further investigated using the HPA database. As shown in Figure 1E,F, the IHC assays were performed by using antibodies CAB019379 and HPA056069, respectively. The protein expression of PSMD7 was higher in HNSCC than in normal head and neck epithelial tissues. These results suggested that PSMD7 was significantly highly expressed in HNSCC than in associated normal tissues.

### PSMD7 is a key gene for survival of HNSCC cells

To determine whether PSMD7 is essential for cell survival of HNSCC, the data of HNSCC cell lines from Project Achilles were explored. CERES dependence score was obtained from Depmap. As shown in Figure 1G, when knocking down of PSMD7 genes with CRISPR-Cas9 system, CERES scores of total 21 HNSCC cell lines were less than −1. These results suggested that PSMD7 was a critical gene for cell survival of HNSCC.

### Association of PSMD7 expression and clinicopathological characteristics

**Figure 2:**
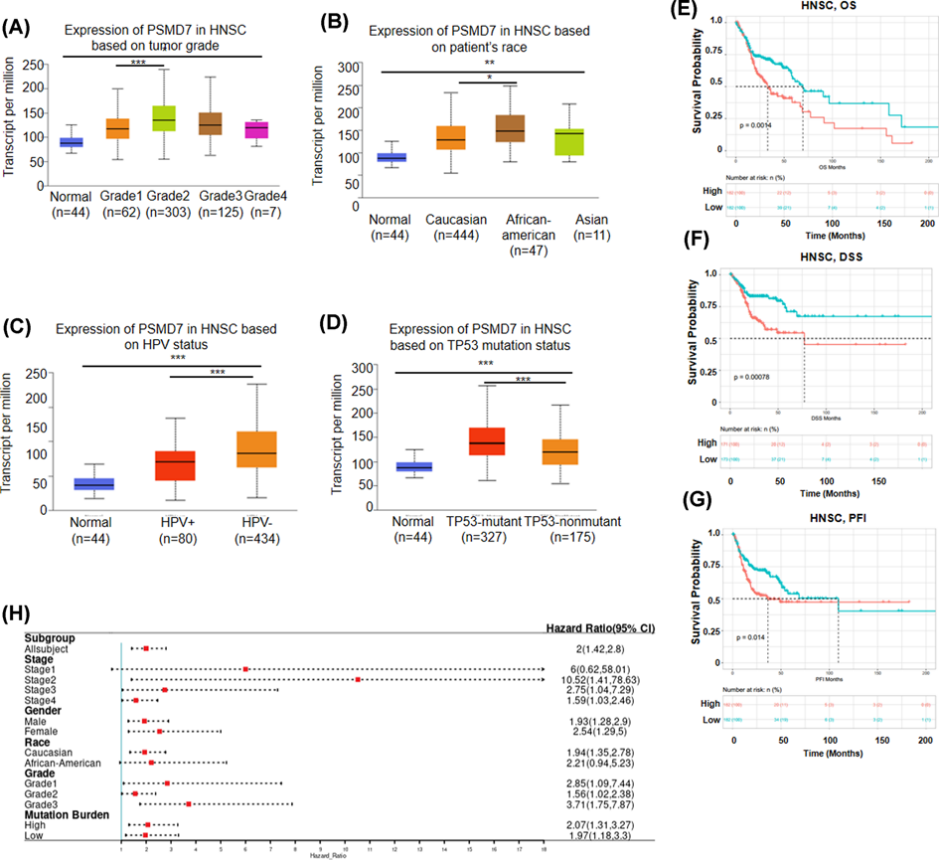
High mRNA expression of PSMD7 indicates poor prognosis in HNSCC patients PSMD7 expression was analyzed in different (**A**) tumor grades (from grade 1 to 4), (**B**) races, (**C**) HPV infection statues and (**D**) TP53 mutation status. Prognostic values of (**E**) OS, (**F**) DSS and (**G**) PFI in HNSCC patients were analyzed based on mRNA expression of PSMD7 using Kaplan–Meier plotter. (**H**) Forest plots showing the association between PSMD7 expression and clinicopathological features in HNSCC patients. *, *P*<0.05; **, *P*<0.01; ***, *P*<0.001.

Because expression of PSMD7 was significantly up-regulated in HNSCC tissues, then the UALCAN was the used database to analyze the mRNA expression profiles of PSMD7 based on clinicopathological parameters. Results of UALCAN database analysis revealed that no statistical expression difference was found in stratified analysis of age, gender, cancer stage and metastasis status groups (Supplementary Figure S2A–D). Regarding the tumor grade, the expression of PSMD7 was significantly increased in grade 2 than in grade 1 (Figure 2A). Expression of PSMD7 was dramatically elevated in Caucasian patients with HNSCC compared with African-American patients (Figure 2B). According to whether patients were infected with human papillomavirus (HPV), expression of PSMD7 was significantly up-regulated in HPV-negative patients than in HPV-positive patients (Figure 2C). Moreover, expression of PSMD7 in TP53-mutant patients was significantly higher than which in TP53-wild-type HNSCC patients (Figure 2D).

### The prognostic evaluation of PSMD7 genes in patients with HNSCC

To determine the association between PSMD7 expression and the prognosis of HNSCC patients, the TCGA-HNSC was investigated using Sangerbox. As shown in Figure 2E,F,G, high expression of PSMD7 was correlated with an unfavorable prognosis of HNSCC patients (OS, *P*=0.0014; DSS, *P*=0.00078; PFI, *P*=0.014).

To further understand the prognostic value of PSMD7 expression in HNSCC, the correlation between the mRNA expression of PSMD7 and OS of HNSCC patients based on clinical features of HNSCC patients was investigated by using the Kaplan–Meier plotter database. As shown in Figure 2F, high expression of PSMD7 suggested an inferior prognosis in all subgroup analysis except for stage 1 and African-American groups (*P*>0.05) .

### Identification of key candidate genes from the PSMD7 interaction network

**Figure 3:**
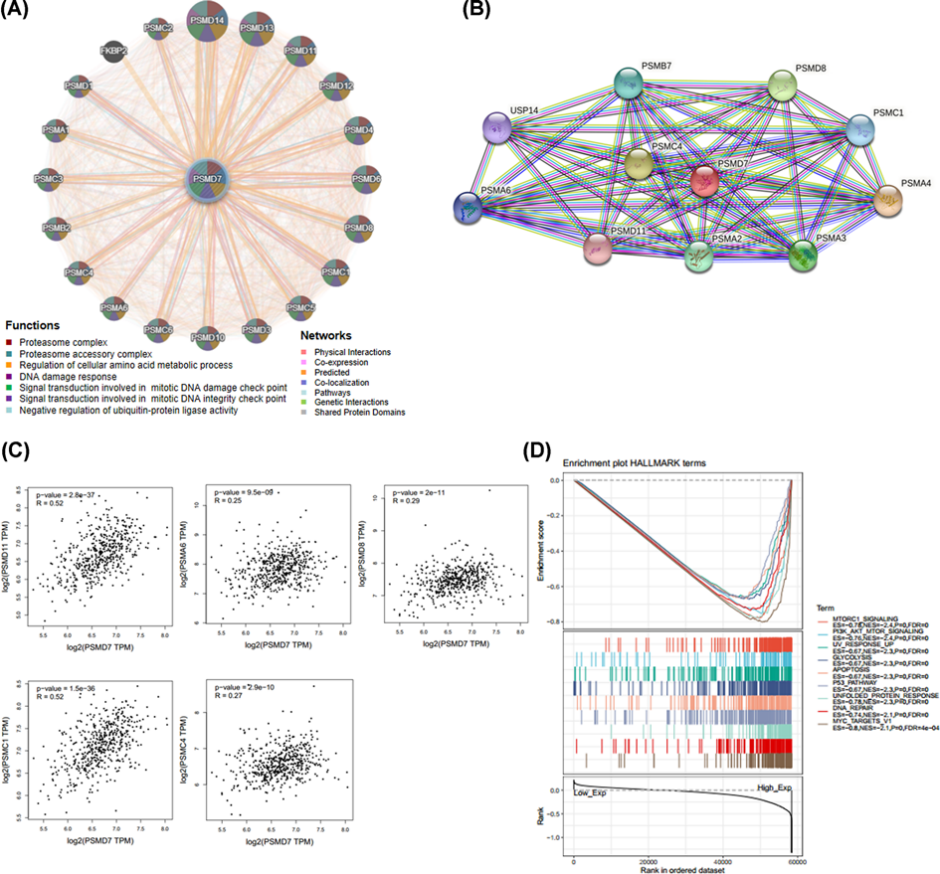
Interaction analysis of PSMD7 at the gene and protein levels (**A**) The gene–gene interaction network for PSMD7was analyzed using the GeneMANIA database. The 20 most frequently changed neighboring genes are shown. Each node represents a gene. The node color represents the possible functions of the respective gene. (**B**) The PPI network, which contained 11 nodes and 38 edges, was constructed using the STRING database. (**C**) Scatterplots of correlations between PSMD7 expression and PSMD11, PSMA6, PSMC1, PSMC4 and PSMD8 in HNSCC. Tumor-associated pathway determined by GSEA analysis based on PSMD7 expression (**D**).

To explore mechanism of PSMD7 in HNSCC and analyzed the function of these genes, a gene–gene interaction network for PSMD7 was constructed using the GeneMANIA database. The hub node representing PSMD7 was surrounded by 20 nodes representing genes that were significantly correlated with PSMD7 (Figure 3A). The tightest associated five genes were PSMD14, PSMD13, PSMD11, PSMD12 and PSMD4. Further functional analysis indicated that the proteins encoded by these genes were significantly linked with the following terms: proteasome complex, proteasome accessory complex, regulation of cellular amino acid metabolic process, DNA damage response, signal transduction involved in mitotic DNA damage check point, signal transduction involved in mitotic DNA integrity check point and negative regulation of ubiquitin-protein ligase activity. To further explore the function of PSMD7, a PPI network was constructed using the STRING database. A total of ten PSMD7-interacting proteins were included in the PPI network complex by filtering (Figure 3B). Importantly, five common hub genes were identified from the GeneMANIA and STRING databases: PSMD11, PSMA6, PSMC1, PSMC4 and PSMD8. The correlation between PSMD7 and these five interacting proteins were determined with the GEPIA database. As shown in Figure 3C, the expression of PSMD7 was strongly correlated with that of PSMD11, PSMA6, PSMC1, PSMC4 and PSMD8 in HNSCC.

### The GSEA of PSMD7 in HNSCC

GSEA was performed to evaluate Hallmark effect gene sets. As shown in Supplementary Table S1, a total of 48 critical pathways were significantly influenced by increased PSMD7 expression in HNSCC. The top five influenced pathways were MTORC1_SIGNALING, UV_RESPONSE_UP, P53_PATHWAY, APOPTOSIS and PI3K_AKT_MTOR_SIGNALING. Several affected pathways were significantly associated with cancer process, growth and metastasis such as MTORC1_SIGNALING, PI3K_AKT_MTOR_SIGNALING and APOPTOSIS (Figure 3D) et al.

### Association between PSMD7 expression and the immune infiltration level in HNSCC

**Figure 4:**
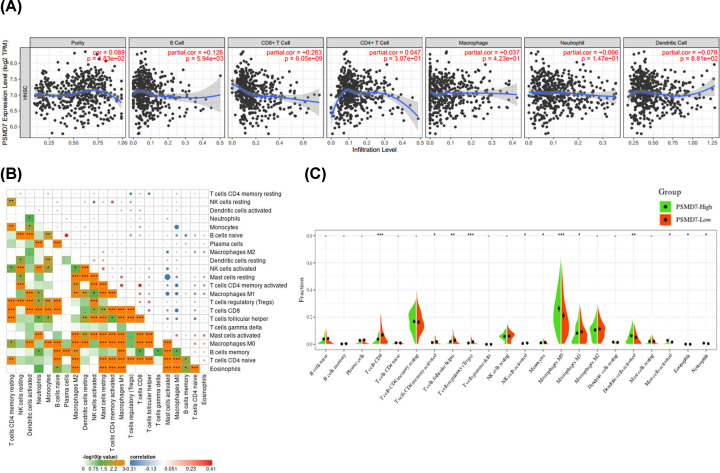
Association of PSMD7 expression with immune infiltration levels in HNSCC (**A**) The association between PSMD7 expression and infiltration levels of B cells, CD4^+^ T cells, CD8^+^ T cells, macrophages, neutrophils and dendritic cells in the TIMER database. (**B**) Pairwise relationship between immune cell abundance ratios. The numerical values represent the correlation value. (**C**) immune cell distribution based on PSMD7 expression was analyzed with the CIBERSORT algorithm.

Tumor-infiltrating lymphocytes are independent predictors of the sentinel lymph node status and cancer survival. Hence, exploring the association between PSMD7 expression and immune infiltration is meaningful. The mRNA expression of PSMD7 in HNSCC was positively associated with tumor purity (Cor = 0.089, *P*=3.83e-02) in the TIMER database. In addition, PSMD7 expression was significantly correlated with the infiltration of B cells (Cor = −0.126, *P*=5.94e-03) and CD8^+^ T cell (Cor = −0.263, *P*=6.25e-09) in HNSCC (Figure 4A). To further investigate the underlying mechanisms of this immune response, the correlation of the 22 immune cells in HNSCC was evaluated using the established computational resource CIBERSORT. The correlation between different immune cells were shown in Figure 4B. To determine the effect of PSMD7 on the tumor microenvironment (TME), we analyzed the infiltration level of the 22 immune cells in high expression of PSMD7 tissues compared with low expression of PSMD7 tissues. As shown in Figure 4C, the infiltration levels of CD8 T cells, activated CD4 T cell, T cells follicular helper, Tregs, resting NK cells, monocytes and M1 macrophages were significantly increased in PSMD7-Low expression group. However, M0 macrophages, activated dendritic cells, activated mast cells, eosinophils and neutrophils were found higher ratios in PSMD7-high expression tissues. To further examined the results, the TISIDB database including 28 tumor-infiltrating lymphocytes in HNSCC was analyzed. Totally, 24 out of 28 tumor-infiltrating lymphocytes (Th1 cells, activated B cells, activated CD8 T cells, eosinophils, Tfh cells, CD56 bright cells, macrophages, activated CD4 cells, NKT cells, Th2 cells, Tem CD8 cells, Th17 cells, MDSC cells, Imm B cells, pDC cells, neutrophils, Mem B cells, Tgd cells, NK cells, CD56dim cells, Tem CD4 cells, Tcm CD4 cells, Mast cells, Treg cells) were significantly affected by PSMD7 expression (Supplemental Figure S3A–X). These findings indicated that PSMD7 was strongly correlated with immune infiltration in HNSCC.

### Correlation analysis between PSMD7 expression and immune marker sets

To further investigate the correlation between PSMD7and these various ratios of infiltrating immune cells, the association between PSMD7 expression and immune markers of diverse immune cells was determined using the TIMER database. As shown in Table 1, expression of PSMD7 was significantly associated with immune cell markers both in none and purity tissues. Furthermore, the association between PSMD7 and different types of functional T cells, including Th1, Th1-like, Th2, Treg, resting Treg, effector Treg, effector T cells, naïve, effector memory, resistant memory, and exhausted T cells was also explored in TIMER. As shown in Table 2, the mRNA expression status of PSMD7 was significantly associated with 16 out of 34 T-cell markers in PSMD7 after adjusting for tumor purity (STAT1, IFNG, CD4, STAT3, STAT5B, PDCD1, GZMK, GZMA, ITGAE, CXCR6, MYADM, LAYN, CTLA4, IL7R). These results indicated that PSMD7 expression was significantly associated to immune infiltration in HNSCC, suggesting that PSMD7 played a critical role in immune escape in the HNSCC microenvironment.

**Table 1:** Correlation analysis between PSMD7 and gene markers of immune cells in TIMER

Description	Gene	None		Purity	
		cor	*P*	cor	*P*
**CD8^+^ T cell**	CD8A	−0.269879676	3.66E-10	−0.266733742	1.78E-09
	CD8B	−0.294252161	6.93E-12	−0.291547544	4.09E-11
**T cell (general)**	CD3E	−0.239970527	2.84E-08	−0.235758416	1.18E-07
	CD3D	−0.269516309	3.87E-10	−0.269775888	1.14E-09
	CD2	−0.25639306	2.79E-09	−0.25306339	1.21E-08
**B cell**	CD19	−0.152733585	0.000462	−0.152073472	0.000704733
	CD79A	−0.156279062	0.000338	−0.150649666	0.000791803
**Monocyte**	CD86	−0.022038368	0.615406	−0.012323562	0.78489683
	CSF1R	−0.082908225	0.058365	−0.075993809	0.091893853
**TAM**	CCL2	0.031454162	0.473314	0.033132865	0.462946863
	CD68	0.162673321	0.000189	0.166724694	0.000200369
	IL10	0.031545239	0.472033	0.040729546	0.366832978
**M1**	IRF5	0.011163704	0.799142	0.018163393	0.687461625
	NOS2	−0.031096133	0.478367	−0.013854787	0.758949909
	PTGS2	0.185600202	1.98E-05	0.215615102	1.35E-06
**M2**	CD163	0.003115116	0.943396	0.007176506	0.873714692
	VSIG4	0.021585108	0.622693	0.015849173	0.725560225
	MS4A4A	−0.01446873	0.741554	−0.01213203	0.788159886
**Neutrophils**	CEACAM8	−0.079666313	0.068959	−0.071350028	0.11359861
	ITGAM	−0.054647302	0.212587	−0.040833696	0.36560788
	CCR7	−0.15333211	0.000439	−0.148845175	0.000916451
**NK cell**	KIR2DL1	−0.080743273	0.065277	−0.082057233	0.068696254
	KIR2DL3	−0.177847761	4.38E-05	−0.172263032	0.000121083
	KIR2DL4	−0.184411203	2.24E-05	−0.189621916	2.25E-05
	KIR3DL1	−0.205599256	2.17E-06	−0.19664461	1.09E-05
	KIR3DL3	−0.135682133	0.00189	−0.133129102	0.003060098
	KIR3DL2	−0.182043383	2.86E-05	−0.174512023	9.82E-05
	KIR2DS4	−0.125220666	0.004165	−0.111713423	0.013067763
**Dendritic cell**	HLA-DPB1	−0.199999831	4.12E-06	−0.202559302	5.81E-06
	HLA-DQB1	−0.14360475	0.001001	−0.144081885	0.001337689
	HLA-DRA	−0.146263653	0.000803	−0.146456344	0.001109397
	HLA-DPA1	−0.143039026	0.001049	−0.145819199	0.001166842
	CD1C	−0.042418931	0.333409	−0.043104753	0.339529507
	NRP1	0.125649268	0.004037	0.120435388	0.007427088
	ITGAX	−0.02406102	0.583355	−0.006710017	0.881861364

**Table 2:** Correlation analysis between PSMD7 and gene markers of different types of T cells in TIMER

Description	Gene	None		Purity	
		cor	*P*	cor	*P*
**Th1**	STAT4	−0.10114	0.020828	−0.18974	0.105415
	STAT1	0.008769	0.841578	0.414054	0.000277
	IFNG	−0.26887	2.44E-27	−0.14561	0.030088
	TNF	0.039576	0.366843	0.107362	0.362574
**Th1-like**	CXCL13	−0.11846	0.000125	−0.07836	0.343824
	HAVCR2	−0.08445	0.006331	−0.00235	0.977341
	BHLHE40	0.050577	0.2487	0.227605	0.051337
	IFNG	−0.26887	2.44E-27	−0.14561	0.030088
	CD4	−0.12025	0.005945	−0.29028	0.012365
	IL21	−0.1736	6.69E-05	NA	NA
**Th17**	STAT3	0.064025	0.144073	0.406324	0.000367
	IL17A	−0.11502	0.008529	0.049882	0.672983
**Treg**	FOXP3	−0.09476	0.000173	−0.0833	0.216339
	CCR8	−0.00251	0.935358	−0.09255	0.263223
	STAT5B	0.133412	0.002255	0.264717	0.022925
	TGFB1	0.269326	3.99E-10	−0.00487	0.967154
**Effctor T cell**	FGFBP2	0.198862	4.69E-06	−0.16171	0.168671
	FCGR3A	−0.05773	0.187906	−0.00179	0.98798
**Naïve T cell**	SELL	−0.11694	0.000152	−0.07531	0.363005
	TCF7	0.040145	0.359996	0.08508	0.470237
**Effective memory T cell**	PDCD1	−0.26233	1.16E-09	−0.28918	0.012457
	DUSP4	−0.04858	0.267856	0.208797	0.074305
	GZMK	−0.26276	1.09E-09	−0.28185	0.014981
	GZMA	−0.24293	1.89E-08	−0.31584	0.006323
	IFNG	−0.26887	2.44E-27	−0.14561	0.030088
**Resistant memory T cell**	CD69	−0.09034	0.039081	−0.14743	0.209552
	ITGAE	0.087335	0.046109	0.310892	0.00723
	CXCR6	−0.21241	9.71E-07	−0.30702	0.007797
	MYADM	0.26734	5.41E-10	0.429989	0.000152
**Exhausted T cell**	TIGIT	−0.18092	3.21E-05	−0.1546	0.188451
	LAYN	0.261621	1.29E-09	0.276387	0.017407
	HAVCR2	−0.08445	0.006331	−0.00235	0.977341
	IL2RA	−0.05368	0.220779	−0.08552	0.468786
	CTLA4	−0.18753	1.61E-05	−0.28331	0.014446
	TNFRSF9	−0.03975	0.364795	0.219394	0.060365
**General memory T cell**	IL7R	0.154781	0.000386	0.386611	0.000668
	SELL	−0.11694	0.000152	−0.07531	0.363005

### Prognostic analysis of PSMD7 expression based on immune cell infiltration in HNSCC

**Figure 5:**
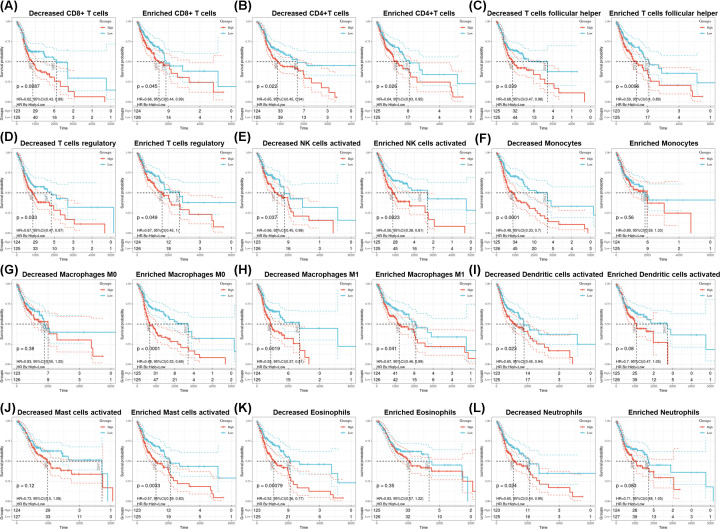
Prognostic analysis of the high and low PSMD7 expression based on immune cell subgroups in HNSCC (**A**–**L**) Correlations between PSMD37 in different immune cell subgroups and OS in HNSCC patients were estimated by the CIBERSORT algorithm.

Because PSMD7 expression was significantly correlated with immune infiltration and poor prognosis in HNSCC patients, whether expression of PSMD7 affected the prognosis of HNSCC patients via its effects on immune infiltration was also investigated. As shown in Figure 5, high expression of PSMD7 was significantly associated with poor prognosis in the HNSCC patient cohorts with all Tregs, all activated NK cell, decreased monocytes, enriched M0 macrophages, all M1 macrophages, all activated dendritic cells, enriched activated mast cells, decreased eosinophils and decreased neutrophils. These findings indicated that high PSMD7 expression may influence prognosis of HNSCC patients partially through immune infiltration.

## Discussion

Protein ubiquitination is a post-translational modification (PTM) that involves the reversible attachment of ubiquitin to amino acid side chains, most commonly a lysine, on the target protein [17]. The ubiquitinproteasome system becomes reversible through the action of deubiquitinating enzymes (DUBs) in a process that can edit and cleave monomeric modifiers such as ubiquitin from substrates [18]. Most ubiquitinated proteins undergo proteasomal degradation by 26S proteasome complex as a macromolecule, which is involved in cell cycle progression, apoptosis, transcription, DNA repair, protein quality control, and antigen presentation [19,20]. PSMD7 and PSMD14 are the core components of the 26S proteasome and are closely connected [12]. PSMD7 interacts with PSMD14 to activate proteasome function to regulate ubiquitinated substrate degradation. Many studies have revealed that PSMD14 is an antiproteasome target for tumor therapies of different cancer types [21–23]. However, the role and function of PSMD7 in tumorigenesis remains largely unknown.

In the present study, the expression of PSMD7 in HNSCC tissues and relative normal tissues was investigated. The results obtained from the TCGA, Sangerbox, UALCAN and Oncomine databases indicated that the mRNA expression of PSMD7 is significantly increased in HNSCC. PSMD7 expression was correlated with various clinicopathological features, including tumor grade, population, HPV infection and TP53 mutation. The association between PSMD7 expression and the prognosis of HNSCC patients was evaluated by using the CVCDAP databases. High expression of PSMD7 was significantly associated with poor prognostic outcomes, including OS, DSS and PFI. Increased PSMD7 influenced several key pathway associated with carcinogenesis and cancer process via GSEA. Importantly, knockdown of PSMD7 significantly inhibited HNSCC cells growth. Finally, PSMD7 was strongly correlated with immune infiltration and influenced the prognosis of HNSCC patients.

Zhao et al. demonstrated that PSMD7 level was significantly up-regulated in breast cancer tissues using IHC analysis, and high expression of PSMD7 was associated with poor survival of patients with breast cancer [16]. Down-33regulating of PSMD7 significantly inhibited cells proliferation of breast cell lines [16]. Similar with the findings, Shi et al. has reported that knockdown of PSMD7-induced ESCC apoptosis via a caspase-3 dependent pathway and decreased cells proliferation [15]. Thus, we performed a pan-cancer analysis to demonstrate the expression profiles and prognostic values of PSMD7 in various cancers. Similarly, our findings indicated that PSMD7 was found highly expressed in both BC and ESCC tissues than in corresponding normal tissues, and increased PSMD7 suggested an inferior prognosis in patients with BC or ESCC (results not shown). Then, we focus on the association between PSMD7 and HNSCC. We observed that the role of PSMD7 in HNSCC was consistent with which in BC and ESCC. Importantly, high expressed PSMD7 was associated with several tumor-relative pathways, including PI3K_AKT_mTOR, P53, KRAS, angiogenesis et al. These results suggested that PSMD7 may promote the occurrence and development of HNSCC. Through ionizing intracellular macromolecules, such as DNA, and by indirectly producing reactive oxygen species (ROS), such as the hydroxyl radical and superoxide, radiation causes DNA damage [24]. Well-organized DNA repair mechanisms were composed of various repair proteins and sensors, which rapidly response to these lesions, repair DNA damage and maintain genomic integrity [25]. As the subunit of 26S proteasome, PSMD7 was found to be decreased after 36 h irradiation using MS-MS method [26]. The inhibitory effect of both low- and high-dose irradiation on proteasome activity was consistent with a previous publication [27]. Interestingly, our findings suggested that PSMD7 high expression was positively associated with DNA repair pathway, which suggested a potential mechanism that radiation may damage activity of DNA repair through PSMD7-dependent manner.

Over the past 10 years, cancer immunotherapy has made a monumental breakthrough in multiple cancer types and has been gradually been applied to clinical cancer care, which also rejuvenated the study of tumor immunology [28]. By blocking programmed death-1 (PD-1)/programmed death-ligand 1 (PD-L1) and cytotoxic T-lymphocyte-associated antigen 4 (CTLA-4), cancer immunotherapy has achieved remarkable therapeutic progress in various kinds of malignances, including HNSCC [29,30]. Immune infiltration in the TME plays an important role in cancer development and occurrence and to influence clinical outcomes of patients with malignances [29,31]. Since immune cells are the cellular basis of immunotherapy, a deep understanding of immune infiltration in the TME is critical to reveal the potential molecular mechanisms and provide new immunotherapeutic strategies to improve clinical curative effect [32,33]. Mitochondria also play a key role in inflammation, immunity and the TME in coping with extraneous infection, stress and damage [34,35]. Mitochondrial dynamics may affect immune cell polarization and inflammatory responses [36,37]. Toll-like receptor-regulated switching to chondriokinesis in tumor-associated macrophages (TAMs) leads to T-44cell activation and enhanced anti-tumor immunity [36]. In the present study, high expression of PSMD7 was associated with activating of various human immune cells, including CD4^+^ T cell, CD8^+^ T cells, Tregs et al. Intron retention (IR) is one of the major forms of alternative splicing in eukaryotes. Recently, a study has reported that the expression of PSMD7 predominantly regulated by IR during both human and mouse CD4^+^ T cell activation, which was consistent with our findings [38]. Furthermore, high expression of PSMD7 indicates an inferior prognosis based on different immune infiltration. These results suggest that PSMD7 acts as an important role in immune infiltration, and could be a potential prognostic marker.

The findings in the present study improved our understanding of the correlation between PSMD7 and HNSCC, but some limitations still existed. First, although the mRNA and protein expression of PSMD7 in HNSCC and relative normal tissues were validated based on data from multiple public databases, further experiments are needed to uncover the protein expression profiles of PSMD7 in HNSCC and molecular mechanisms associated with how PSMD7 regulates growth of tumor cell and tumor-infiltrating cells to influence the prognosis of HNSCC patients. Second, the correlation between PSMD7 expression and prognosis of HNSCC patients was not strong. Lacking more large public datasets in the survival analysis limited the precise of last conclusion, especially in some little cohorts. Third, this study performed bioinformatics analysis on only PSMD7. It is hardly to distinguish if the PSMD7 dominates the variation of associated pathway and DEGs. More experimental evidences are needed to further explore their relationships.

In summary, PSMD7 expression is increased in HNSCC and significantly correlated with the clinicopathologic stages and prognosis of HNSCC patients. PSMD7 and associated pathway changes are involved in the HNSCC growth and development. Moreover, PSMD7 expression is significantly correlated with the level of immune cell infiltration. Thus, PSMD7 likely could be a prognostic marker and has a potential effect on immunotherapy in HNSCC.

## Supplementary Material

Supplementary Figures S1-S3 and Table S1

## Data Availability

The data that support the findings of our study are openly available from cBioportal (http://www.cbioportal.org/), Sangerbox (http://sangerbox.com/), The Kaplan–Meier plotter (www.kmplot.com), UALCAN (http://ualcan.path.uab.edu), Oncomine (http://www.oncomine.org), HPA (https://www.proteinatlas.org/), GSEA (http://www.broad.mit.edu/gsea), GeneMANIA (http://www.genemania.org), STRING (https://string-db.org/), Depmap portal (https://depmap.org/portal), TIMER (https://cistrome.shinyapps.io/timer/), CIBERSORT (https://cibersort.stanford.edu/) and TISIDB (http://cis.hku.hk/TISIDB/).

## Abbreviations

CTLA-4: cytotoxic T-lymphocyte-associated antigen 4
DSS: disease-specific survival
ESCC: esophageal squamous cell carcinoma
GSEA: gene set enrichment analysis
HNSCC: head and neck squamous cell carcinoma
HPA: Human Protein Atlas
HPV: human papillomavirus
IHC: immunohistochemistry
IR: intron retention
NK: natural killer
OS: overall survival
PD-1: programmed death-1
PD-L1: programmed death-ligand 1
PFI: progression-free interval
PSMD7: proteasome 26S subunit, non-ATPase 7
PTM: post-translational modification
ROS: reactive oxygen species
TAMs: tumor-associated macrophages
TCGA: The Cancer Genome Atlas
TIMER: Tumor Immune Estimation Resource
TME: tumor microenvironment
