# Supplementary material for: Identification of PSMD7 as a prognostic factor correlated with immune infiltration in head and neck squamous cell carcinoma
Source: Biosci Rep. 2021 Mar 24;41(3):BSR20203829. doi: 10.1042/BSR20203829 (PMC7990087; doi:10.1042/BSR20203829)
Supplement: Supplementary Figures S1-S3 and Table S1 [file BSR-2020-3829_supp.pdf]

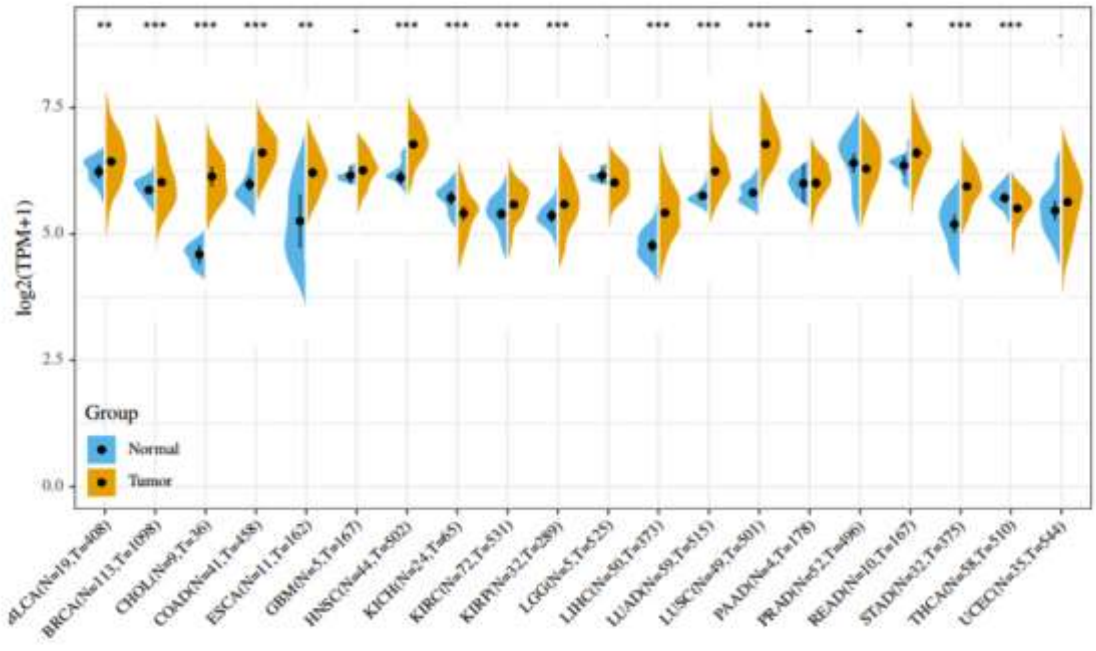

Supplemental Figure 1. The transcription level of PSMD7 in different human cancers. PSMD7 expression levels in various types of cancer in the Sangerbox database.

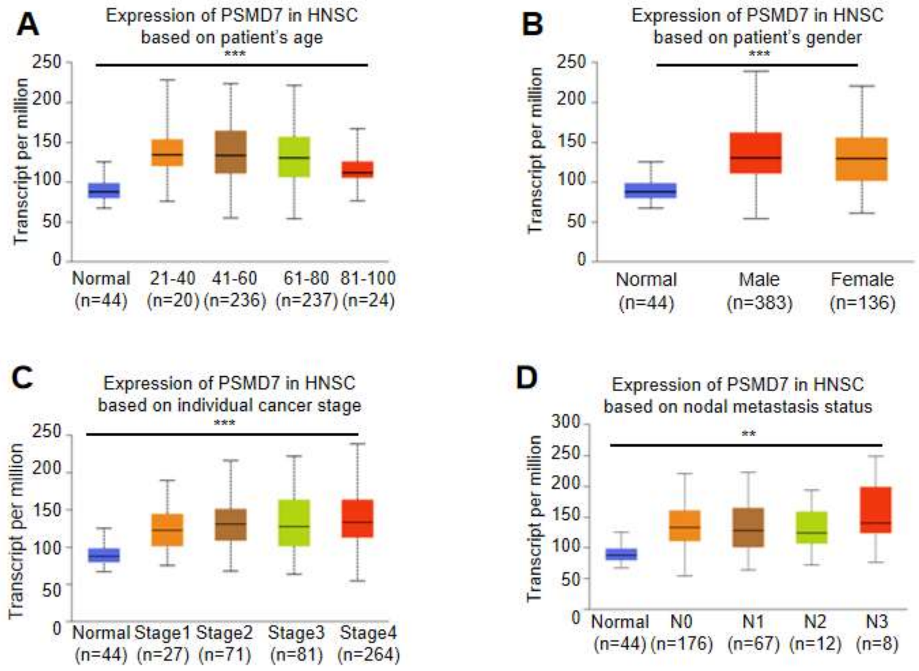

Supplemental Figure 2. Expression ofPSMD7 based on clinical pathological parameters in HNSCC patients in the UALCAN database. PSMD7 expression was analyzed in (A)different ages (from 21 to 100 years), (B) male and female HNSCC patients, (C) cancer clinicalstages of HNSCC (from stage 1 to stage 4) and (D) different nodal metastasis statuses (from N0 to N3). *, P<0.05; **, P<0.01; ***, P<0.001; PSMD7, Proteasome 26S subunit, non-ATPase 7; HNSCC, head and neck squamous-cell carcinoma;

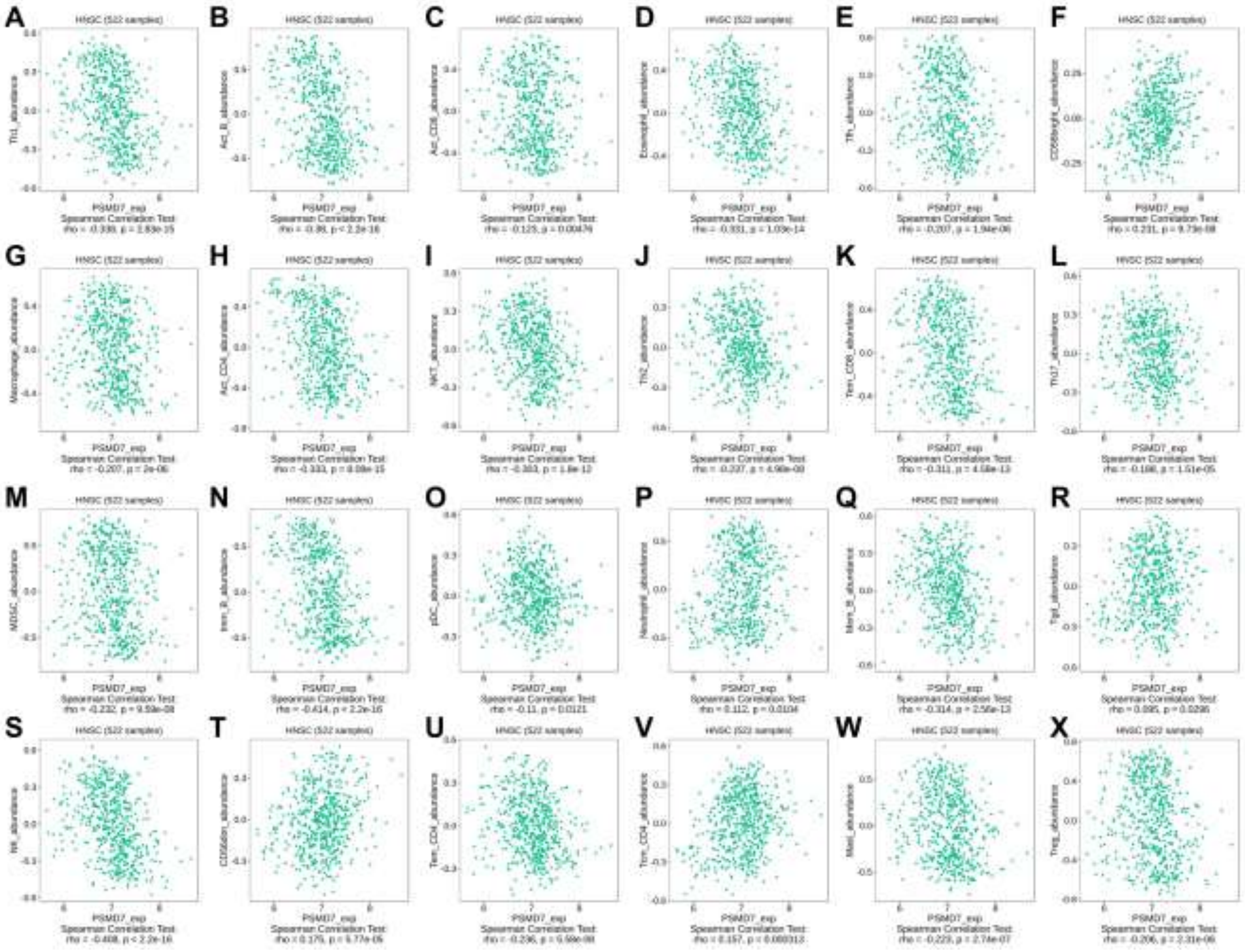

Supplemental Figure 3. PSMD7 expression was associated immune celldistribution with TISIDB analysis in HNSCC. (A-X) Positive relationship was found between different immune cell distribution and PSMD7 expression. PSMD7, Proteasome 26S subunit, non-ATPase 7; HNSCC, head and neck squamous-cell carcinoma

Supplemental Table 1. GSEA Analysis of DEGs based on PSMD7 high expression.

NAME	SIZE	ES	NES	NOM p-val	FDR q-val
HALLMARK_MTORC1_SIGNALING	198	-0.78207	-2.3837	0	0
HALLMARK_UV_RESPONSE_UP	156	-0.66582	-2.35985	0	0
HALLMARK_P53_PATHWAY	197	-0.66642	-2.31532	0	0
HALLMARK_APOPTOSIS	160	-0.67221	-2.28029	0	0
HALLMARK_PI3K_AKT_MTOR_SIGNALING	104	-0.75971	-2.27722	0	0
HALLMARK_UNFOLDED_PROTEIN_RESPON	110	-0.78359	-2.26838	0	0
HALLMARK_GLYCOLYSIS	197	-0.67428	-2.23088	0	0
HALLMARK_HEME_METABOLISM	197	-0.66872	-2.22864	0	0
HALLMARK_PEROXISOME	104	-0.67557	-2.17367	0	0
HALLMARK_ANDROGEN_RESPONSE	96	-0.70589	-2.15798	0	0
HALLMARK_ESTROGEN_RESPONSE_LATE	197	-0.5808	-2.14828	0	0
HALLMARK_DNA_REPAIR	148	-0.73819	-2.14168	0	0
HALLMARK_APICAL_JUNCTION	194	-0.65058	-2.13926	0	0
HALLMARK_PROTEIN_SECRETION	95	-0.80144	-2.12546	0	0
HALLMARK_MYC_TARGETS_V1	197	-0.80407	-2.1069	0	0
HALLMARK_ADIPOGENESIS	194	-0.66715	-2.10544	0	0
HALLMARK_MYC_TARGETS_V2	58	-0.82682	-2.10115	0	0
HALLMARK_MITOTIC_SPINDLE	198	-0.7809	-2.09593	0	6.46E-05
HALLMARK_ESTROGEN_RESPONSE_EARLY	195	-0.59677	-2.08646	0	1.19E-04
HALLMARK_TNFA_SIGNALING_VIA_NFKB	198	-0.64711	-2.0835	0	1.13E-04
HALLMARK_IL2_STAT5_SIGNALING	196	-0.61867	-2.07302	0	1.07E-04
HALLMARK_HYPOXIA	191	-0.6038	-2.06813	0	1.55E-04
HALLMARK_REACTIVE_OXYGEN_SPECIES	47	-0.70712	-2.05963	0	1.94E-04
HALLMARK_XENOBIOTIC_METABOLISM	199	-0.57102	-2.05719	0	2.31E-04
HALLMARK_TGF_BETA_SIGNALING	54	-0.76793	-2.05353	0	2.22E-04
HALLMARK_G2M_CHECKPOINT	192	-0.77734	-2.04095	0	2.61E-04
HALLMARK_CHOLESTEROL_HOMEOSTASIS	74	-0.65079	-2.03679	0	3.41E-04
HALLMARK_SPERMATOGENESIS	135	-0.55301	-1.98281	0	0.001236
HALLMARK_UV_RESPONSE_DN	137	-0.67848	-1.98233	0	0.001194
HALLMARK_COMPLEMENT	199	-0.59433	-1.96862	0.00396	0.001311
HALLMARK_INFLAMMATORY_RESPONSE	198	-0.61558	-1.9663	0.008163	0.001302
HALLMARK_FATTY_ACID_METABOLISM	157	-0.6024	-1.95466	0	0.001527
HALLMARK_OXIDATIVE_PHOSPHORYLATI	184	-0.68995	-1.92536	0	0.002187
HALLMARK_KRAS_SIGNALING_UP	195	-0.5582	-1.92081	0	0.0023
HALLMARK_WNT_BETA_CATENIN_SIGNAL	42	-0.68214	-1.91791	0	0.002331
HALLMARK_E2F_TARGETS	197	-0.76037	-1.91689	0	0.002266
HALLMARK_NOTCH_SIGNALING	32	-0.66385	-1.86747	0.002041	0.003856
HALLMARK_BILE_ACID_METABOLISM	112	-0.54376	-1.84299	0.00207	0.005139
HALLMARK_IL6_JAK_STAT3_SIGNALING	86	-0.59106	-1.81202	0.017578	0.0065
HALLMARK_INTERFERON_GAMMA_RESPON	199	-0.65129	-1.79723	0.024793	0.007539
HALLMARK_HEDGEHOG_SIGNALING	35	-0.6102	-1.7791	0.005906	0.008907
HALLMARK_APICAL_SURFACE	43	-0.52892	-1.7744	0.002041	0.008993
HALLMARK_ANGIOGENESIS	36	-0.61792	-1.6956	0.026052	0.017864

NOM p-val < 0.05 and FDR q-val < 0.25 were considered as significant
Abbreviations: DEG, difference expression of gene; GSEA, gene set enrichment analysis; PSMD7, Proteasome 26S subunit, non-ATPase 7
